# The effect of alpha-linolenic acid supplementation on ADHD symptoms in children: a randomized controlled double-blind study

**DOI:** 10.3389/fnhum.2014.00780

**Published:** 2014-10-07

**Authors:** Gal Dubnov-Raz, Zaher Khoury, Ilana Wright, Raanan Raz, Itai Berger

**Affiliations:** ^1^Lifestyle, Exercise and Nutrition Clinic, The Edmond and Lily Safra Children’s Hospital, Sheba Medical CenterTel Hashomer, Israel; ^2^Sackler Faculty of Medicine, Tel Aviv UniversityTel Aviv, Israel; ^3^Faculty of Medicine, Hadassah Medical Center – Hebrew UniversityJerusalem, Israel; ^4^Leumit Medical ServicesNetanya, Israel; ^5^Department of Environmental Health, Harvard School of Public HealthBoston, MA, USA; ^6^The Neuro-Cognitive Center, Pediatric Division, Hadassah Medical Center – Hebrew UniversityJerusalem, Israel

**Keywords:** fatty Acids, omega-3, attention, hyperactivity, ADHD, linolenic acids

## Abstract

**Background:** Attention deficit-hyperactivity disorder (ADHD) is the most common neuro-developmental disorder in childhood. Its pharmacologic treatment mostly includes methylphenidate, yet many parents seek alternative, “natural,” therapeutic options, commonly omega-3 fatty acids. Previous studies of supplementation with fish oil or long-chain omega-3 fatty acids to children with ADHD yielded mixed results. The use of alpha-linolenic acid (ALA), a medium-chained, plant-based omega-3 fatty acid (18:3 n-3), has not been sufficiently examined in this population.

**Methods:** Forty untreated children with ADHD, aged 6–16 years, were randomized to receive either 2 g/day of oil containing 1 g ALA or placebo, for 8 weeks. Before and after supplementation, the children underwent a physician assessment of ADHD symptoms and a computerized continuous performance functions test. The children’s parents and teachers filled out Conners’ and DSM questionnaires.

**Results:** Seventeen (42.5%) children completed the study, eight in the supplementation group, nine in the placebo group. Main drop-out reasons were capsule size, poor compliance, and a sense of lack of effect. No significant difference was found in any of the measured variables tested before and after supplementation, in both study groups. No between-group difference was found in the changes of the various measures of ADHD symptoms throughout the study period.

**Conclusion:** Supplementation of 2 g/day of oil containing 1 g ALA did not significantly reduce symptoms in children with ADHD. Future studies in this field should consider an alternative method to deliver the oil, a higher dose, and a larger sample size.

## INTRODUCTION

Attention-deficit hyperactivity disorder (ADHD) is a childhood onset disorder with a relatively high global prevalence, ranging from 2.2–17.8% (Skounti et al., 2007). ADHD is considered the most common neuro-behavioral disorder of childhood, and one of the most prevalent chronic health conditions affecting school-aged children (Wolraich et al., 2011).

The pharmacologic treatment of ADHD mostly includes methylphenidate (Wolraich et al., 2011). Surprisingly, one study from Israel found that the prevalence of methylphenidate prescription use in the population was only 2.5% – a much smaller rate than the estimated prevalence of ADHD in Israel (Vinker et al., 2006). A possible explanation as to why a large proportion of children with ADHD are not adequately treated could be the stigmatization of methylphenidate. Indeed, many parents continuously seek alternative, “natural,” therapeutic options other than methylphenidate (Berger et al., 2008). Omega-3 fatty acids are among the most common dietary supplements used in children with ADHD. The basis for this treatment stems from studies that identified low levels of omega-3 fatty acids in plasma phospholipids or red blood cell membranes of children with ADHD (Mitchell et al., 1987; Stevens et al., 1995; Antalis et al., 2006).

Several trials of supplementation with fish oil or long-chain omega-3 fatty acids [mostly eicosapentaenoic acid (EPA) and docosahexaenoic acid (DHA)] to children with ADHD have been conducted, and yielded mixed results. Systematic reviews and meta-analyses on this topic summarized that there is currently no consensus that omega-3 fatty acids influence ADHD symptoms (Richardson, 2006; Chalon, 2009; Raz and Gabis, 2009; Bloch and Qawasmi, 2011; Gillies et al., 2012). One possible mechanism suggested for the common lack of effect is a relatively poor incorporation of these fatty acids into the brain. In a pioneering study, Vaisman et al. (2008) showed that consumption of EPA and DHA that were incorporated into phospholipids, resulted in higher circulating levels and better executive functioning in children with ADHD, as compared with fish oil. The authors identified significant correlations between the chemical changes and the clinical effects. Another supplementation study with long chain omega-3 fatty acids conjugated to phosphatidylserine, in order to improve their absorption, also showed improved behavior of children with ADHD (Manor et al., 2012).

Fewer trials examined the effect of the plant-based alpha-linolenic acid (ALA), the parent medium-chain omega-3 fatty acid, on ADHD symptoms. In one published study, 200 mg ALA administered as flaxseed oil together with vitamin C were given to children with ADHD (Joshi et al., 2006). The authors noted increased levels of circulating EPA and DHA, with improvements in parent-rated ADHD symptoms of impulsivity, restlessness, inattention, and self-control. Yet the lack of control group in this study, the relying on parent report only, and the concomitant addition of vitamin C, prevent from drawing clear conclusions on the effect of ALA alone. In another study, children with ADHD received either a supplement containing 120 mg of ALA and 480 mg of linoleic acid and, or vitamin C as placebo, for 7 weeks (Raz et al., 2009). Treatment effects were measured using questionnaires and a computerized test of attention, and did not differ significantly between groups. Of note, these authors administered a relatively low dose of ALA, while also supplementing with a higher dose of the parent fatty acid of the omega-6 family which could inhibit conversion of ALA to the long chain omega-3 fatty acids through competition on the same enzymes. It still remains unknown whether supplementation of a higher dose of ALA could affect ADHD symptoms more than placebo.

The aim of the current study was to examine if supplementation with an ALA-rich sage oil can improve symptoms in children and adolescents diagnosed with ADHD.

## MATERIALS AND METHODS

### PARTICIPANTS

The study population included 40 children and adolescents aged 6–16 years, recently diagnosed with ADHD, who were drug naïve and untreated, from two ambulatory ADHD specialty clinics in Israel. Exclusion criteria were refusal to undergo any or all of the testing procedures or to take the designated supplement; a history of chronic health conditions other than ADHD; or use of any chronic medications or dietary supplements, specifically methylphenidate or fatty acid/fish oil supplements. The study was approved by the Institutional Review Board of Hadassah Medical Center, Jerusalem, Israel, conducted according to the Declaration of Helsinki, and registered in a clinical trials registry before recruitment (#NCT00874536). At least one parent signed an informed consent form, and each participant verbally agreed to participate.

### INTERVENTION

The study participants were randomized 1:1 to receive either 2 g/day of sage oil or an identical appearing lactose placebo in gel capsules. Participants were instructed to consume two gel capsules daily. The composition of sage oil varies slightly by crop year, and is 50–54% ALA, 20–23% oleic acid, 16–18% linoleic acid, 6–7% palmitic acid, and 2–3% stearic acid (Tulukcu et al., 2012). This corresponds to a supplementation of 1 g/day of ALA, a dose that had been shown previously in adults to elevate the concentrations of omega-3 fatty acids (Barceló-Coblijn et al., 2008). The supplementation period lasted 8 weeks.

### RANDOMIZATION

Both types of capsules were supplied in identical amounts in solid plastic bottles. The bottles were numbered consecutively and coded by a person uninvolved in the study, and each participant received three bottles that contained all pills necessary for the study duration. Each ADHD clinic received half of the bottles, numbered consecutively. The children that agreed to participate in the study received their designated bottles in consecutive order. All study participants, parents, teachers, and study personnel were blinded to the allocation until completion of all data collection.

### OUTCOMES AND DATA COLLECTION

The primary outcomes at study end were ADHD symptoms, as assessed by validated questionnaires and a computerized continuous performance test (CPT, see below). Each child met the criteria for ADHD according to Diagnostic and Statistical Manual of Mental Disorders (DSM)-IV-TR criteria (American Psychiatric Association, 2000), as assessed by a physician certified in ADHD diagnosis and treatment.

The diagnostic procedure included an interview with the child and parents, filing of DSM-based ADHD diagnostic questionnaires, and a medical and neurological examination. Parents and teachers filled the appropriate Conners’ rating scales (Conners, 1997a,b).

We used the MOXO-CPT, which is a standardized computerized test designed to diagnose ADHD-related symptoms (Berger et al., 2009). The total duration of the test is 15 min, and it is composed of eight levels of 53 trials each. In each trial, a stimulus in the form of a cartoon picture (designated as “target” or “non-target”) is presented for 500, 1000, or 3000 ms, followed by a “void” period of the same duration, during which the tested individual should respond by pressing the keyboard “space” bar as quickly as possible. In each level, 33 target and 20 non-target stimuli are presented. The tested participant is instructed not to respond to any other stimuli except the target, and not to press any other key but the space bar, and only once. The response timing and accuracy is measured after each stimulus. The test includes distracting stimuli that are presented to the tested participant. These distractors are short animated video clips, containing visual, and auditory features. Six different distractors are included, each of them could appear as only visual, only auditory, or as a combination of both. Each distractor is presented for a different duration ranging from 3.5–14.8 s, with a fixed interval of 0.5 s between two distractors. Distractor onset is not synchronized with target/non-target onset, and could therefore appear either during the stimulus events or during the void period. The burden of the distracting stimuli increases during the test.

The MOXO-CPT measures four performance indices: attention, timing, impulsivity, and hyperactivity. The attention index corresponds to the number of correct space bar keystrokes in response to a target stimulus. This index is considered a pure measure of sustained attention, because it measures correct responses independently of the response time. The timing index is the number of correct responses given quickly, still within the period that the target stimulus is present on screen. The impulsivity index is the number of responses performed following a non-target stimulus. The hyperactivity index is the total number of commission responses that are not coded as impulsive responses (e.g., multiple keystrokes in response to a target stimulus, responses performed in the void period after a non-target stimulus, random key pressing). Other measures of the MOXO-CPT are described in detail elsewhere (Berger et al., 2013; Cassuto et al., 2013).

The same assessment tools were used after 8 weeks of supplementation in both groups. At this final assessment visit, missing pills in the bottles were counted, to assess adherence.

### STATISTICAL ANALYSIS

The differences in questionnaire scores and CPT results between baseline to end-of-study values within each group were compared by the Wilcoxon signed-ranks test. The differences in clinical data, questionnaire scores, and CPT results between study groups at baseline and at trial end were compared by the Mann–Whitney *U* Test. The between-group differences in the changes of questionnaire scores and CPT results were also compared by the Mann–Whitney *U* Test. Proportions were compared using Fisher’s exact test. Statistical significance was defined as a two-sided *p*-value <0.05.

## RESULTS

Twenty children were included in each group, and received their designated supplement. After 8 weeks, only 17 participants remained in the study, and underwent the post-supplementation assessment: nine in placebo group (six males, three females, mean age 10.9 ± 2.3 years) and eight in the oil group (four males, four females, mean age 11.1 ± 3.0 years). Reasons for dropout were difficulty to take the capsules due to size or taste (*n*=7), a subjective sense of lack of effect (*n* = 4), loss of contact (*n* = 5), or lack of interest to perform the second assessment (*n* = 7). None of the participants complained about the most common intolerance to fish oils, namely a “fishy” smell and aftertaste.

### PARENT QUESTIONNAIRES

**Table 1** presents data from parent questionnaires, regarding their perception of their children’s behavior, before and after the supplementation period. There were significant differences between study groups at baseline in two of the three scales, indicating worse ADHD behavior in the omega-3 group. There were also significant differences between groups at trial end in all scales, again in the direction of worse behavior. However, there were no significant between-group differences in the changes from pre- to post- supplementation values in any of the measured parameters.

**Table 1:** Data from parent Conners’ questionnaires at baseline and study end in both groups.

	Placebo (*n* = 9)			Omega-3 (*n* = 8)			*p*-value between changes***
	Pre	Post	*p-*value	Pre	Post	*p*-value	
Parent Conners’ ADHD Index	62 [47–70]	62 [46–64]	0.19	76* [71–90]	79** [54–89]	0.68	0.79
Parent Conners’ Global Index	68 [49–80]	71 [42–76]	0.50	85* [78–90]	77** [72–90]	0.46	0.42
Parent Conners’ DSM-IV: total	65 [50–79]	62 [45–69]	0.04	78 [63–89]	87** [64–90]	0.89	0.22

Data is presented as median and [range].

*Significantly different (*p*< 0.05) from the corresponding baseline value of the placebo group.

**Significantly different (*p*< 0.05) from the corresponding post-supplementation value of the placebo group.

***p-value of the comparison between the pre-post changes in each group.

### TEACHER QUESTIONNAIRES

**Table 2** presents data from teacher questionnaires, regarding their perception of the children’s classroom behavior, before and after the supplementation period. There were no significant differences between study groups at baseline or at trial end in any of the tested parameters. Additionally, there were no significant between-group differences in the changes from pre- to post- supplementation values in any of the measures.

**Table 2:** Data from teacher Conners’ questionnaires at baseline and study end in both groups.

	Placebo (*n* = 9)			Omega-3 (*n* = 8)			*p-*value between changes*
	Pre	Post	*p*-value	Pre	Post	*p-*value	
Teacher Conners’ ADHD index	59 [59–75]	61 [59–69]	0.27	69 [53–89]	69 [58–87]	0.67	0.26
Teacher Conners’ Global Index	63 [60–64]	64 [60–85]	0.65	65 [58–68]	72 [56–90]	0.25	0.76
Teacher Conners’ DSM-IV: total	62 [57–75]	61 [57–67]	0.18	63 [46-90]	66 [56–90]	0.28	0.17

Data is presented as median and [range].

*p-value of the comparison between the pre-post changes in each group.

### DSM criteria

Scores of DSM-criteria questionnaires also did not differ between placebo and omega-3 groups. Respective scores at baseline were 11 (range 6–17) and 13 (range 6–17), *p* = 0.29, with no measurable change in any of the values at trial end [11 (range 6–17) and 13 (range 6–17), *p* = 0.47].

### COMPUTERIZED CONTINUOUS PERFORMANCE TEST

**Table 3** presents data from the MOXO-CPT that the children undertook before and after the supplementation period. There were no significant differences between study groups at baseline or at trial end in any of the tested parameters. Additionally, there were no significant between-group differences in the changes from pre- to post- supplementation values in any of the measures.

**Table 3:** Data from the MOXO performance test at baseline and study end in both groups.

	Placebo (*n* = 9)			Omega-3 (*n* = 8)			*p-*value between changes*
Measure	Pre	Post	*p*-value	Pre	Post	*p*-value	
Timing	167 [114–253]	158 [54–208]	0.87	125 [23–194]	178 [130–214]	0.14	0.20
Reaction time	0.47 [0.39–0.60]	0.54 [0.10–0.65]	0.50	0.54 [0.05–0.67]	0.52 [0.42–0.66]	0.89	0.88
Impulsivity	14 [2–130]	7 [1–96]	0.73	7 [0–23]	21 [5–157]	0.14	0.20
Attention	230 [166–264]	244 [58–263]	1.00	178 [24–258]	242 [196–256]	0.22	0.53
Hyperactivity	48 [7–293]	29 [0–435]	0.50	20 [4–151]	61 [10–569]	0.35	0.27

Data is presented as median and [range].

*p-value of the comparison between the pre-post changes in each group.

## DISCUSSION

The aim of this study was to examine if supplementation with an oil rich in ALA can improve behavior and function in children and adolescents with ADHD. Using several validated questionnaires and a CPT, we found no evidence for a significant effect in any direction. Although we did identify significantly higher post-supplementation scores in the parent Conners’ questionnaires in the omega group, these were attributed to baseline differences; there were no significant differences between the changes in these questionnaire scores between groups. A decrease in the parent Conners’ DSM-IV questionnaire score was seen in the placebo group only, with no other index of improvement in any other measure in this group. We believe that this does not truly reflect clinical improvement, and is probably a random finding resulting from multiple comparisons.

Several previous studies of fish oil/long chain omega-3 supplementation to children with ADHD have been performed, with recent meta-analyses showing no significant clinical effect (Bloch and Qawasmi, 2011; Gillies et al., 2012). It should be noted that one meta-analysis on this topic did identify a small, statistically significant effect – but which is clinically much lesser than that obtained by methylphenidate and other medications (Bloch and Qawasmi, 2011). Therefore, from a clinical point of view, there is currently no evidence to support choosing omega-3 fatty acids over methylphenidate for ADHD treatment. The authors explained the discrepancy between their own findings and the null effect seen in most individual trials, by the small sample sizes used in the single studies. They calculated that in order to obtain sufficient statistical power to identify the small effect of omega-3 in ADHD compared to placebo, clinical trials would require a sample of approximately 330 children. The authors stated that the omega-3 fatty acid supplementation trials examining childhood ADHD used 26–117 participants only. Conversely, the Cochrane meta-analyses (Gillies et al., 2012), which included 13 trials with 1011 participants overall, concluded that there is little evidence that omega-3 fatty acid supplementation provides any benefit for the symptoms of ADHD in children and adolescents.

To our knowledge, only two studies that utilized plant-based omega-3 fatty acids in ADHD were published to-date (Joshi et al., 2006; Raz et al., 2009). Both used relatively small amounts of ALA (200 and 120 mg, respectively). In the study by Joshi et al. (2006), increased levels of circulating EPA and DHA were found, demonstrating that even a much lower dose than that used in our study (yet when combined with vitamin C), is sufficient to increase concentrations of omega-3 fatty acids in the body. The improvement seen in most measures in that study, such as total hyperactivity score, restlessness, impulsiveness, and inattentiveness, was very large, around 1 SD. Nevertheless, the lack of placebo-control group and the addition of vitamin C, prevent from isolating the individual effect of ALA *per se*. Collectively, there is still a wide gap in our knowledge regarding the effects of omega-3 fatty acids in ADHD, which is even greater in the field of plant-based ALA. The main reasons are the large variations in the types of fatty acids used (omega-3 with or without omega-6, fish- or plant-based oils, etc.), in their dosages, in the proportions between EPA and DHA, and in the different characteristics and numbers of participants in each study.

We recognize that our trial has several limitations. Firstly, we had a relatively small sample size with a high dropout rate, attributed both to the reported inconvenience of consuming 2 g of oil per day, and to the subjective feeling of a lack of effect by the participants or their parents. Recruiting a larger sample size of newly diagnosed, non-medicated children for such a trial may be a challenge, as many parents do request an immediate and efficient treatment once their child is initially diagnosed with ADHD. Secondly, we chose a wide age range of 6.5–16 years, which might have caused a wide variation in test results, especially in the computerized test. Baseline data of the DSM questionnaire also showed a wide range of disturbance, as reflected by the range of DSM scores seen. We *a priori* chose a wide age range and included participants from both sexes in order to increase generalizability, yet eventually this might have been a drawback. Lastly, the dose used might have still been too low to increase the amount of brain EPA and DHA. While this dose has been shown to elevate omega-3 fatty acids concentrations in red blood cells in adults (Barceló-Coblijn et al., 2008), we would expect at least the same effect in children and adolescents; however, examining the brain content of these fatty acids in children following supplementation is currently not possible.

The strengths of the study were its randomized, placebo-controlled design; the choice of non-medicated, otherwise healthy children; and the concomitant use of several validated tools that directly assessed the child, as well as parent and teacher perceptions of his behavior.

In summary, in this study, supplementation of 1 g/day of ALA using an ALA-rich oil to children and adolescents with ADHD did not improve any behavioral measure, as tested by several validated questionnaires and a computerized CPT. We do acknowledge that a major limitation to the trial was the relatively small sample size, attributed to a relatively high dropout rate. Nevertheless, our findings are in concert with many other studies which used both ALA and long-chain omega-3 fatty acids. Given the high dropout rate, and in light of previous research and anticipated effect size, we recommend recruiting much larger numbers of participants, and possibly using a higher ALA dose in future similar studies.

## AUTHOR CONTRIBUTIONS

Gal Dubnov-Raz contributed to study design, conductance, data analysis, and drafted the first manuscript. Zaher Khoury contributed to study conductance and data analysis. Ilana Wright and Itai Berger contributed to study design, conductance, and data analysis. Raanan Raz contributed to study design and data analysis. All authors contributed significantly to the manuscript text, and approved its final version.

## Conflict of Interest Statement

The Associate Editor Yael Leitner declares that, despite being affiliated to the same institution as author Gal Dubnov-Raz, the review process was handled objectively and no conflict of interest exists. Itai Berger serves on the scientific advisory board of Neuro-Tech Solutions Ltd. All other authors declare no conflicts of interest.
